# Use of Probiotics as Prophylaxis for Postoperative Infections

**DOI:** 10.3390/nu3050604

**Published:** 2011-05-12

**Authors:** Bengt Jeppsson, Peter Mangell, Henrik Thorlacius

**Affiliations:** Department of Surgery, Lund University and Skane University Hospital, Malmo, SE 205 02, Sweden; Email: peter.mangell@med.lu.se (P.M.); henrik.thorlacius@med.lu.se (H.T.)

**Keywords:** probiotics, postoperative infections, immune function, bacterial translocation, gastrointestinal surgery, liver transplantation

## Abstract

Postoperative bacterial infections are common despite prophylactic administration of antibiotics. The wide-spread use of antibiotics in patients has contributed to the emergence of multiresistant bacteria. A restricted use of antibiotics must be followed in most clinical situations. In surgical patients there are several reasons for an altered microbial flora in the gut in combination with an altered barrier function leading to an enhanced inflammatory response to surgery. Several experimental and clinical studies have shown that probiotics (mainly lactobacilli) may reduce the number of potentially pathogenia bacteria (PPM) and restore a deranged barrier function. It is therefore of interest to test if these abilities of probiotics can be utilized in preoperative prophylaxis. These factors may be corrected by perioperative administration of probiotics in addition to antibiotics. Fourteen randomized clinical trials have been presented in which the effect of such regimens has been tested. It seems that in patients undergoing liver transplantation or elective surgery in the upper gastrointestinal tract prophylactic administration of different probiotic strains in combination with different fibers results in a three-fold reduction in postoperative infections. In parallel there seems to be a reduction in postoperative inflammation, although that has not been studied in a systematic way. The use of similar concepts in colorectal surgery has not been successful in reducing postoperative infections. Reasons for this difference are not obvious. It may be that higher doses of probiotics with longer duration are needed to influence microbiota in the lower gastrointestinal tract or that immune function in colorectal patients may not be as important as in transplantation or surgery in the upper gastrointestinal tract. The favorable results for the use of prophylactic probiotics in some settings warrant further controlled studies to elucidate potential mechanisms, impact on gut microbiota and influence on clinical management. The use of probiotics must be better delineated in relation to type of bacteria, dose and length of administration.

## 1. Introduction

Bacterial infection is a frequent complication following operations in the gastrointestinal tract. Despite prophylactic administration of antibiotics the incidence of postoperative infections ranges from 10-30% in resectional surgery [1]. Most infections are caused by bacteria of enteric origin [2]. In spite of restricted use of prophylactic antibiotics, the emergence of antibiotic resistance has increased significantly [3]. The gut microbial flora and mucosa are also affected by surgical trauma resulting in the gut barrier dysfunction and intestinal microbial imbalance. This may further aggravate systemic inflammation and depress immune function [4]. All these factors contribute to an increased risk of postoperative infections and sepsis. Alternative strategies in preparing patients for gastrointestinal surgery besides mechanical bowel preparation and administration of antibiotics must be considered.

Probiotics (live microorganisms which, when consumed in adequate amounts as part of food, confer a health benefit on the host) are able to influence several of these factors. They can stabilize the intestinal barrier by stimulating epithelial growth, mucus secretion and motility as well as enhance innate immunity by inhibition of IL-10 and stimulation of secretory IgA, neutrophils and reduction of inflammatory cytokines [5]. The effects on IL-10 can be varied with both inhibition and stimulation. Furthermore, administration of probiotics suppresses growth of potentially pathogenic microorganisms, e.g., *E. coli* and Enterobacteriaceae. It has been hypothesized by several authors that these characteristics can be used in a clinical setting of preoperative prophylaxis for reduction of postoperative infections. Preoperative antibiotic prophylaxis constitutes more than ten percent of antibiotic usage in surgery and a reduction could lead to a reduced pressure on development of antibiotic resistance. It may therefore be of interest to study if probiotics may be used in the preoperative preparation of patients undergoing gastrointestinal operations.

In recent years a few randomized trials on the use of prophylactic probiotics in major gastrointestinal surgery mostly for cancer and liver transplantation. Since this is a new concept on the use of probiotics, it is important to critically analyze these studies before a wider application.

## 2. Upper Gastrointestinal Surgery (Table 1)

Two studies from Berlin addressed patients undergoing major resections in the upper gastrointestinal tract. In one study administration of *Lactobacillus plantarum* 299(10^9^) was compared to placebo and heat-killed bacteria [6]. Bacteria were administered enterally 5-7 days before operation. The two groups receiving lactobacilli (live or heat-killed) had a postoperative infection rate of 10% compared to 30% in controls. This difference was statistically significant (*p* < 0.01). The influence on gut microbiote from administration of probiotics was not evaluated. The impact on immune parameters could not be shown since most parameters (NK cells, T cells and lymphocytes) were within normal limits in both study groups.

In the other study, 89 patients undergoing pancreatic resections were included [7]. Patients were randomized to receive synbiotics or placebo starting one day before surgery and continued eight days postoperatively [7]. The synbiotics contained four different lactic acid bacteria and four bioactive fibers: betaglucan, inulin, pectin and resistant starch. In total, 20 g of fiber per day was administered. There was a significant reduction in postoperative surgical site infections from 40% to 12.5% (*p* < 0.05). There was also a shorter hospital stay in the synbiotic group and duration of antibiotic administration was shorter (median 2 *vs.* 10 days). Gut microbiota or effect on immune parameters was not studied. The total dose of bacteria was higher than the previous study [6] and the effect was related to live bacteria, since there was no use of heat-killed bacteria. The authors used a mixture of bacteria and fiber and the observed effect could also partly be ascribed to the fiber content.

In another randomized controlled trial postoperative enteral administration of a synbiotic mixture of 10^10^*Lactobacillus casei* and *Bifidobacterium breve* and 15 g/day of fiber (galactooligosaccharides) was studied [8]. One group received synbiotic treatment before and after operation, while one group only had postoperative treatment. Both groups received antibiotic prophylaxis. When therapy was given both pre- and postperatively there was a significant reduction in postoperative infection rate (12 *vs.* 30%). There was no difference in intestinal permeability measured by Lactulose-Mannitol test. The probiotic bacteria could be identified in feces upon administration. The pre- and postoperative administration resulted in an increase in NK cell activity, number of lymphocytes and IL-6, whereas CRP (C-reactive protein) decreased. The results are impressive, but the study is difficult to evaluate, since there is no proper control [8].

**Table 1:** Results of studies on the use of probiotics/synbiotics as prophylaxis for postoperative infections in elective surgery.

Author	Patients	Comparison	Bacteria, type, doses	Length of admin.	SSI	Impact on flora	Immuno-modulation
Rayes 2002 [6]	Abdom. surgery *n* = 90	Live LB, heat-killed Lb *vs.* placebo	Lb plantarum 299 10^9^	5-7 days	30% *vs.* 10% *vs.* 10%	Not studied	No diff.
Rayes 2007 [7]	Pancreo-duodenectomy *n* = 89	Synbiotics *vs.* placebo	Pediococcus pent Leuconsultic mes Lb paracasei Lb plantarum 10^10^	9 days	12.50% *vs.* 40%	Not studied	Not studied
Sugawara 2006 [8]	Biliary surgery *n* = 81	Synbiotics postop *vs.* synbiotics pre/postop	Lb casel B. breve 10^10^	2 w before *vs.* 2 w after op	30% *vs.* 12.10%	Lb increased	IL-6, WBC increased CRP decreased
McNaught 2002 [9]	Abdom. surgery mostly colon resection *n* = 129	Lb *vs.* placebo	Lb plantarum 299v 5 × 10^7^	1 w before op + postop	13% *vs.* 15%	Lower PPM	Translocation and CRP unchanged
Anderson 2004 [10]	Abdom. surgery *n* = 137	Synbiotics *vs.* placebo	Lb bulgaricus Lb acidophilus B. lactis Streptoccus therm 4 × 10^9^	12 days	32% *vs.* 31%	Not studied	No diff. in bact. translocation CRP, IL-6 and Ig M
Reddy 2006 [11]	Colonic surgery *n* = 88	Placebo *vs.* Neomycin *vs.* Synbiotics + MBP *vs.* Synbiotics − MBP	Lb bulgaricus Lb acidophilus B. lactis Streptoccus therm 4 × 10^9^	?	No diff. between groups 17% overall	Synbiotics + MBP reduced Entero- bacteriacease	Synbiotics MBP reduced bact. translocation
Hovart 2010 [12]	Colon Surgery *n* = 68	Synbiotics *vs.* heat killed bact. *vs.* placebo	Pedioccus pent. Leuconostic mesent Lb paracasei 2362 10^12^	3 days preop	No diff. between groups	Not studied	Higher IL-6 and fibrinogen postop in synbiotic group
Rayes 2000 [13]	Liver transplant. *n* = 95	Selective bowel decontamination *vs.* Lb *vs.* placebo	Lb plantarum 299 10^9^	12 days	48% *vs.* 13% *vs.* 34%	Not studied	WBC decrease
Rayes 2004 [14]	Liver transplant. *n* = 66	Synbiotics *vs.* placebo	Pediacoccus pent Leuconsultic mes. Lb paracasei Lb plantarum 10^10^	15 days	3% *vs.* 48%	Not studied	No diff.
Eguch 2010 [15]	Living donor Liver transplant. *n* = 50	Synbiotics *vs.* placebo	Lb casei B. breve	14 days	4% *vs.* 24%	No diff.	Not studied
Woodard 2009 [16]	Gastric by pass *n* = 41	Probiotics *vs.* control	Lb species 10^8^	6 months	Not studied	Probiotics reduced bacterial over-growth	Not studied

Lb: Lactobacilli; B.: Bifidobacteria; SSI: Surgical site infection.

## 3. Lower Gastrointestinal Surgery (Table 1)

An early study comparing administration of *Lactobacillus plantarum* 299vpre- and postoperatively with placebo did not show any significant difference in postoperative infections [9]. Translocation and CRP levels were unchanged in the two groups. There was a reduction in the number of potentially pathogenic microbes. The amount of lactobacilli administered was, however, rather low (5 × 10^7^), which partly explains the absence of effect on postoperative infections. The study population consisted of both upper and low gastrointestinal urgery, but mostly colorectal resections (82/129). They were not analyzed separately, but the overall postoperative sepsis rate was 13% and 15% for treatment and controls. These figures are well in line with what could be expected in such a study population. 

In another study from the same group, a synbiotic mixture containing four bacteria and fiber was compared to placebo [8]. The administration was started two weeks prior to colonic surgery. The dose was higher than in the previous study [9] (4 × 10^9^), but there was still no effect on postoperative sepsis, bacterial translocation or inflammatory markers [10]. Surprisingly, there was a three-fold increase in postoperative infections in this study compared to the other from the same group with the same type of surgery.

The same research group has made yet another contribution to the field [11]. They randomized 88 patients undergoing colectomy to four groups with 20-24 patients in each group: mechanical bowel preparation alone; with added neomycin; addition of synbiotics; and synbiotics and neomycin but no bowel preparation. The amount of bacteria was 4 × 10^9^. It is not clearly stated how long the symbiotic mixture was administered. Bacterial translocation was determined by culture of mesenteric lymph nodes and intestinal permeability measured by triple sugar test. 

There were no significant alterations of bacterial translocation or intestinal permeability, although bacterial translocation seemed lower in patients with mechanical bowel preparation, neomycin and synbiotics. Similarly, in patients who had all three interventions a significant reduction in Enterobacteriaceae in the colon was observed. These findings did not appear to have any effect on the incidence of postoperative infections and the septic rate is 17% overall with no difference between the groups. This well-performed study is unfortunately limited by the low number of patients. The study was only powered to study impact on translocation.

A newly presented study compared synbiotics, prebiotic with heat-killed bacteria and placebo in patients planned for colonic resection [12]. The same bacterial composition was used as in the two studies referenced as [9] and [10], but a significantly higher amount of bacteria was administered. The study products were given only for three days preoperatively and there seems to be no influence on postoperative wound sepsis, although very little information is given in the paper [12]. It seemed that the postoperative inflammatory response was higher in the synbiotic group, but the reasons for this is not obvious from the study [12]. The paucity of data and the very short length of administration make the contribution of this study to the field of preoperative use of probiotics very limited. 

## 4. Liver Transplantation (Table 1)

One of the earliest presented studies on preoperative use of probiotics was performed in patients undergoing liver transplantation. The transplantation team in Berlin randomized patients undergoing liver transplantation to either selective bowel decontamination, administration of living lactobacilli during 12 days, or placebo containing heat-killed lactobacilli [13]. The probiotic used was *Lactobacillus plantarum* 299 in a reasonable high dose. All patients received prophylactic antibiotics according to local routines. There was a significant reduction in postoperative sepsis and wound infection rate in the group that received living probiotics: 13%*vs.* 34% and 48% with heat-killed lactobacilli and bowel decontamination, respectively. Most infections were cholangitis and pneumonia and the most commonly isolated bacteria was enterococci. They also observed a shorter hospital stay, lower number of days in intensive care and a decreased use of additional antibiotics in the group that received supplementation of lactobacilli [13]. Postoperative leukocyte count was lower in the lactobacilli group. The results of this study are impressive but mechanisms underlying the observed effects could not be clarified. No evaluation of intestinal mucosal floras was done.

The same research group presented another study on liver transplantation patients [14]. In this study, patients were randomized to receive either synbiotics in high amounts, administered during 15 days, or placebo without bacteria. The postoperative infection rate was 3% and 48% respectively, which is highly significant and in line with the previous study. Additional use of antibiotics was similarly reduced. No cultures of intestinal content were done. The reason for the striking reduction in postoperative infections is not clear. There did not seem to be any influence on cellular immune parameters in any group. 

A newly published study addressed similar questions in patients undergoing living donor liver transplantation [15]. In these patients postoperative infectious complications are as frequent as in patients undergoing whole liver transplantation. All patients received a synbiotic mixture starting two days before transplantation and continued two weeks following transplantation. A commercially available mixture was used containing *Lactobacillus casei* and *Bifidobacterium breve*. Controls received placebo. Postoperative sepsis was significantly reduced from 24% to 4%. Most infections were caused by *Enterococcus* spp. and methicillin-resistant *Staphylococcus aureus.* Bacterial profiles in fecal cultures were normalized post-transplant in the synbiotic group compared to controls. 

Immune parameters were not analyzed [15].

The results of this study are in line with the other two studies and support the use of probiotics in future studies in these patients.

## 5. Bariatric Surgery (Table 1)

In patients undergoing gastric by pass surgery for morbid obesity, there is a risk that the enteric microflora may be altered resulting in bacterial overgrowth in the small bowel [17]. This results in nausea, flatulence, diarrhea and abdominal pain. Long-term administration of antibiotics does not seem reasonable to avoid these problems. Another solution could be the use of probiotics. In one recent study, 44 patients undergoing gastric by-pass surgery were randomized to probiotics or placebo [16]. Patients in the probiotic group were given a six month supplement of a commercially available mixture of *Lactobacillus* species. Bacterial overgrowth was evaluated by hydrogen breath test. 

The probiotic group demonstrated significantly fewer signs of bacterial overgrowth than the control group [16]. The breath test was performed at several time points during the study and the effect on bacterial overgrowth was checked after three months on probiotics. Unfortunately no cultures of aspirate from the intestines were done so a qualitative evaluation of floral changes could not be carried out, and the effects of the breath test could be related to other factors than administration of live bacteria.

The indication for use of probiotics in patients with gastric by-pass is further supported by a recent experimental study reporting a relationship between microflora and energy expenditure [18]. Therefore probiotics may safely improve gastrointestinal symptoms after by gastric by-pass surgery including a potential weight loss.

## 6. Discussion

There seems to be striking differences in effects of prophylactic administration of probiotics in different surgical patients. The results relating to reduction in postoperative infections are convincing when used in patients undergoing surgery in the upper gastrointestinal tract and liver transplantation. The use in patients undergoing colorectal surgery does not seem to show any benefit. It is difficult to compare studies performed in different patient groups, since the design of studies is very different both in relation to type of bacteria/fiber, dose and length of administration. Since the results of many controlled trials point in the same direction, one can speculate that the conditions for this therapy are very different in specific parts of the gastrointestinal tract. The small bowel and liver harbor a large part of the immune system and maybe the effect of immune modulation play a greater role in surgery in liver, pancreas and biliary tract as well as oesophagus and stomach. The bacterial content in these organs is relatively low or absent and therefore it may be easier to influence the microbial balance by administration of probiotics and fiber. 

The conditions for use of probiotics in colon and rectum may, on the other hand, be different. In these organs the immune cells play a less prominent role and the number of mucosa-associated bacteria is far greater. This infers that immune modulation probably plays a minor role in colorectal surgery and that it is more important to have an impact on the microbiota. Thus, much higher doses and longer duration of administration of probiotics may be required to detect such an effect.

Although clinical results related to reduction of infections are evident in several studies, few attempts have been made to elucidate potential mechanisms. There are some indications that the inflammatory response to surgery is reduced [8,13]. Potential reduction of bacterial translocation was studied in a few reports and results are inconsistent [9,10,11] and effect is minor, if present at all. The major problems with all these studies are the lack of proper evaluation of mucosal microbiota. Although most authors claim the potential effect of probiotics on gut microbiota, the absence of proper analysis makes it difficult to evaluate the clinical use of probiotics as prophylaxis of postoperative infections. The administered probiotics could be retrieved in one study [8] and reduction of potentially pathogenia bacteria (PPM) as a result of bacterial antagonism was observed in some studies [9,10,18]. The relation of these results to reduction of postoperative infections has not been analyzed. Future studies in this field must more clearly address these issues. The length of administration, dose and type of probiotics used must be clarified, as well as how impact of probiotics on gut microbiota can be evaluated in patients. Furthermore, most studies imply that observed effects relate to the action of live bacteria, although heat-killed bacteria may infer effects as well. This has been addressed in only two studies [6,12], where heat-killed bacteria did not seem to have an impact on postoperative infections. Most authors in the cited studies infer direct effects by live probiotics on gut microbiota as possible mechanism of action, but since the contribution of altered microflora and immune function to postoperative infections is not clear, the issue on live*vs.* heat-killed bacteria must be the focus in future studies.

Administration of probiotics is in general considered safe and many of them are used in different products commercially available to the public. They have been used for many years and reports of side effects have been very few. However, when live bacterial are administered to patients, safety must be carefully monitored. Patients with depression of immune function may be more susceptible to complications than healthy individuals. Although few reports on sepsis induced by administration of probiotics exist, this possibility must be carefully monitored. Furthermore, recent reports indicating increased morbidity and mortality when used in patients with severe pancreatitis also highlight safety concerns. These groups of patients with an ongoing severe inflammation are probably different from patients undergoing elective operations. The results of controlled trials performed so far indicate therapy with probiotics seems safe and without any reported serious side-effects. Further studies are warranted to elucidate potential mechanisms of action in order to optimize its use. 
